# Mechanical Performances of Lightweight Sandwich Structures Produced by Material Extrusion-Based Additive Manufacturing

**DOI:** 10.3390/polym12081740

**Published:** 2020-08-04

**Authors:** Sebastian Marian Zaharia, Larisa Anamaria Enescu, Mihai Alin Pop

**Affiliations:** 1 Department of Manufacturing Engineering, Transilvania University of Brasov, 500036 Brasov, Romania; larisa.enescu@student.unitbv.ro; 2 Department of Materials Science, Transilvania University of Brasov, 500036 Brasov, Romania; mihai.pop@unitbv.ro

**Keywords:** fused filament fabrication, lightweight sandwich structures, mechanical testing, finite element analysis, wing leading edge

## Abstract

Material Extrusion-Based Additive Manufacturing Process (ME-AMP) via Fused Filament Fabrication (FFF) offers a higher geometric flexibility than conventional technologies to fabricate thermoplastic lightweight sandwich structures. This study used polylactic acid/polyhydroxyalkanoate (PLA/PHA) biodegradable material and a 3D printer to manufacture lightweight sandwich structures with honeycomb, diamond-celled and corrugated core shapes as a single part. In this paper, compression, three-point bending and tensile tests were performed to evaluate the performance of lightweight sandwich structures with different core topologies. In addition, the main failure modes of the sandwich structures subjected to mechanical tests were evaluated. The main failure modes that were observed from mechanical tests of the sandwich structure were the following: face yielding, face wrinkling, core/skin debonding. Elasto-plastic finite element analysis allowed predicting the global behavior of the structure and stressing distribution in the elements of lightweight sandwich structures. The comparison between the results of bending experiments and finite element analyses indicated acceptable similarity in terms of failure behavior and force reactions. Finally, the three honeycomb, diamond-celled and corrugated core typologies were used in the leading edge of the wing and were impact tested and the results created favorable premises for using such structures on aircraft models and helicopter blade structures.

## 1. Introduction

Lightweight sandwich structures are used in automotive, sustainable energy, aerospace, marine, and building industries due to their high flexural stiffness-to-weight ratio, excellent thermal insulation and high-energy absorption capacity [1]. The frequent use in engineering applications of lightweight sandwich structures results in better acceleration and lower fuel consumption (for aircraft), but also lower lifecycle costs because of lower operation costs for many applications [2]. The classic methods of manufacturing and assembling of lightweight sandwich structures involve many stages that make production expensive and require the purchase of complex and sometimes difficult to use devices [3].

A modern method of manufacturing lightweight sandwich structures, made from plastic, composite and metallic materials, is represented by additive manufacturing technology. Additive manufacturing technology comprises a wide range of technologies (Stereolithography, Inkjet printing, Fused Filament Fabrication, Selective Laser Sintering, Selective Laser Melting) and consists of material deposition, layer by layer, having as a main purpose the transformation of digital models into physical objects [4]. 

Fused Filament Fabrication (FFF) technology is the most widely used additive manufacturing technology because it can use a wide range of materials, it is quiet and safe, it can produce usable objects and components, the price of 3D printers is affordable and the technology is easy to use [5]. The complex geometries and structures which otherwise are difficult to achieve by using traditional methods can be performed with the help of FFF technology. Additionally, using the FFF technology product prototypes can be created, tested and quickly modified, if needed.

Research regarding the manufacture of lightweight sandwich structures using FFF technology can be divided into several main directions: numerical testing and analysis of the core structures; 3D printing and analysis of gyroid cellular lattice structures; manufacture of sandwich structures by bonding composite material sheets (Carbon Fiber Reinforced Polymers - CFRP) with the core manufactured by additive technologies and 3D printing of sandwich structures for mechanical tests. The honeycomb structures manufactured by additive technologies have been highly researched regarding their compression strength [6], their energy absorption [7] and the shape recovery effect of a 3D-printed structure [8]. Recent studies have highlighted the diversity of the numerical analyses [9] and the analysis of the failures, under compression tests [10] of the honeycomb structures manufactured by FFF technology. In addition, FFF technology has been used to create different cellular topologies, such as Gyroid and Schwarz P [11] or a 3D-printed hierarchical gyroid structure with embedded TiO_2_ nanoparticles [12]. Because the printing of lightweight sandwich structures is difficult, the manufacture of these structures consists of the core printed from different materials, such as polylactic acid (PLA) [13,14], FLX 9795-DM [15], ABS (Acrylonitrile Butadiene Styrene) [14,16] and the skins made from carbon fiber reinforced polymer and they are assembled using the vacuum bagging method. 

These lightweight sandwich structures have been tested for mechanical performance (tensile, three-point bending test and free vibration analysis) resulting in good behavior that facilitates their use in various applications, such as aerospace, transport, civil or military. Different 3D-printed polymeric meta-sandwich structures made of cubic, octet and isomax cellular cores have been used for the development of architectural materials [1,17]. In these studies, the failure modes, mechanical performances and energy absorption of 3D-printed lightweight sandwich panels with architected cellular cores were evaluated. Further, researchers [18] have focused their studies on understanding the influence of different sandwich structures (with three different core shapes, hexagon, triangle, and square shapes) taking into account the following parameters: nature of core shape, number of infill shapes, and orientation of cores and the way the dynamic behavior of sandwich structures is influenced. Preliminary studies investigated the manufacture and testing of mechanical performance (bending and tensile) of honeycomb core sandwich structures [19,20,21], printed with different materials (PLA and ABS) and using different filling techniques. An initial investigation into the manufacture of continuous carbon fiber sandwich structures without the addition of supports has been carried out [22]. In this study, it was demonstrated that continuous carbon fiber 3D printers can be used to flexibly design core shapes that satisfy the desired strength and stiffness. An innovative study was developed by a group of researchers [23] who manufactured a honeycomb structure by FFF printing using thermoplastic shape memory polymer.

However, following the study of the state of present knowledge it can be stated that there are scientific challenges of an interdisciplinary nature that can be exploited, generating original methodologies in the field of testing the mechanical performances of sandwich structures manufactured by FFF technology. The main difficulty in 3D printing of honeycomb sandwich structures is that the upper skin is manufactured above the core spaces; therefore, support structures are needed for good quality 3D printing. When making sandwich structures, the honeycomb core becomes a closed space and the supporting material cannot be removed later. Thus, it results in a sandwich structure with an addition of material which causes a significant increase in its weight.

In this paper, lightweight sandwich structures on the edge (in the XZ plane), without support, were designed and manufactured by FFF technology with three configurations of core: honeycomb, diamond-celled and corrugated. This study also evaluated the mechanical performance of lightweight sandwich structures by conducting mechanical tests (compression, tensile, and three-point bending). After determining the mechanical characteristics, these types of lightweight sandwich structures were introduced in the leading-edge of an airplane wing, in order to determine their feasibility and impact characteristics. In the end, a comparative study was performed between the values of the reaction forces that appeared when breaking the specimens tested for three-point bending and the values of the reaction forces that appeared in the structure of the supports of the FEA (Finite Element Analysis) model.

## 2. Materials and Methods

### 2.1. Design of Sandwich Structures

**Figure 1:**
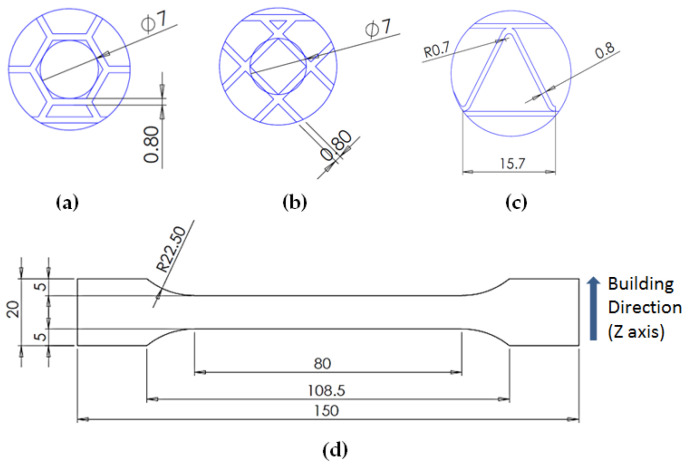
Specimens characteristics (dimensions in mm): (**a**) honeycomb core; (**b**) diamond-celled core; (**c**) corrugated core; (**d**) tensile specimens’ dimensions.

Taking into account the current standards (MIL-STD-401B and ASTM C393) applied to lightweight sandwich structures, the specimens (Table 1) specific to the compression, tensile and bending stresses were designed using the SolidWorks 2016 software. The compression-tested specimens were designed in accordance with current regulations and have the following characteristics: length 50 mm, width 50 cm, height 15 mm and a thickness of the skin of 0.75 mm. For tensile and three-point bending tests, the specimens have the following dimensions: length 150 mm, width 20 cm, thickness specimen 15 mm and thickness of the skin of 0.75 mm. The core typologies that were used in the tested specimens showed the following dimensions: honeycomb core (Figure 1a), diamond-celled core (Figure 1b) and corrugated core (Figure 1c). The specimens specific to the tensile tests have a classic configuration with fillet radius and a solid area for good grip on the test machine (Figure 1d).

### 2.2. Design of Wing Leading Edges

In aviation, sandwich structures are used in the structure of the wings, on their leading-edge. The sandwich structures follow the outline of the aerodynamic profile of the wing in order to stiffen the wing. The modelling of the leading-edge of the wing starting with the introduction of the NACA (National Advisory Committee for Aeronautics) 0018 aerodynamic airfoil coordinates (Table 2).

The leading edge of the wing has the same configuration but with three different core types: honeycomb, diamond-celled, corrugated. The dimension of the skins is 1.5 mm, and the distance between the two skins where the honeycomb, diamond-celled, corrugated core structure was introduced, was 10 mm, keeping the same cell core dimensions as those in Figure 1.

### 2.3. Materials Properties and Manufacturing Conditions

The specimens were manufactured using the BCN3D Sigma printer (Barcelona, Spain) for PLA/ polyhydroxyalkanoate (PHA) material; this material is completely biodegradable. The PLA/PHA mixture is relatively inexpensive and has a higher stiffness compared to the PLA material. The mechanical and thermal properties of the PLA/PHA filament, provided by the manufacturer—FKuR Kunststoff GmbH (Willich, Germany)—were presented in Table 3.

BCN3D Sigma is a high-quality 3D printer, characterized by its innovative dual extrusion approach, with a construction volume of 210 mm × 297 mm × 210 mm. The 3D printing parameters of the specimens are described in Table 4.

**Figure 2:**
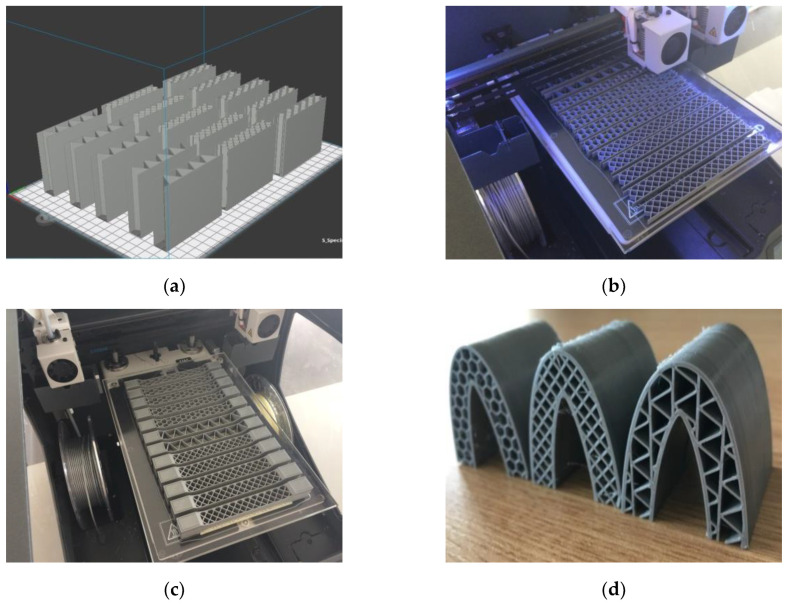
The manufacturing process: (**a**) compression tested specimens; (**b**) three-point bending tested specimens; (**c**) tensile tested specimens; (**d**) the leading edge specimens.

Normally, a sandwich structure consists of two skins and a core and the material from which the core is made can be the same or different from that of the skins. In the case of the sandwich structures made by additive technologies, the material used for the core and skins was the same (PLA/PHA), with the manufacture being done in a single stage, as studied in this work; alternatively the core and skin may be made from different materials, in a situation in which the skins will be later attached to the core. The BCN3D Cura printer software converts the digital model into a set of instructions for the 3D printer and with it the manufacturing parameters have been set. The manufacture of the specimens was carried out without material support (the samples subjected to compression tests (Figure 2a) and the specimens subjected to three-point bending (Figure 2b), with the exception of tensile specimens (Figure 2c), due to their geometry, which required the use of PVA (Polyvinyl Alcohol) support material which is water soluble.

In the manufacturing process of the specimens there were no problems, and their quality and accuracy were high, without any layer debonding. The technological process of additive manufacturing of the leading edge of the wing (Figure 2d) has the same parameters of additive manufacturing and the same material (PLA/PHA), as those used in the specimens of sandwich structures.

### 2.4. Mechanical Testing

#### 2.4.1. Compression Tests

Compression, tensile and three-point bending testing, of sandwich structure specimens was performed on the WDW-150S universal testing machine (Jinan Testing Equipment IE Corporation, Jinan, China). For testing the sandwich structures, 5 specimens were made for each configuration of the core of the structure (honeycomb, diamond-celled, corrugated), depending on the type of test the specimen will be subjected to. Thus, 15 specimens were manufactured for each category of tests, including compression, three-point bending and tensile, totaling 45 specimens.

**Figure 3:**
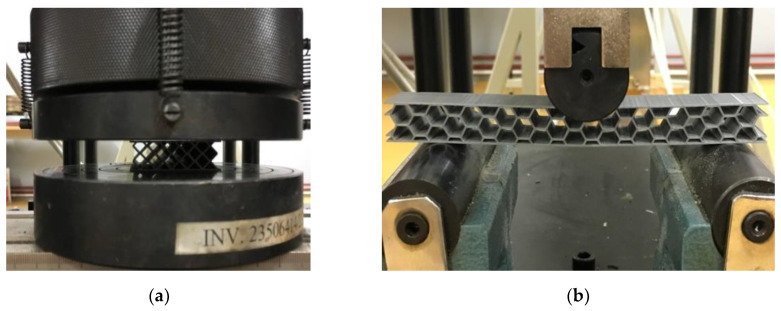
The mechanical tests: (**a**) compression testing of diamond-celled core sandwich specimens; (**b**) three-point bending testing of honeycomb core sandwich structures.

The mechanical compression tests of the lightweight sandwich structures were performed on the WDW-150S mechanical test machine (Figure 3a). The compression tests were performed with a crosshead speed of 5 mm/min. Five tests were performed for each of the lightweight sandwich structures studied (honeycomb, diamond-celled, corrugated). 

Equation (1) and Equation (2) were used to determine the compressive strength (*σ*_c_) and compressive modulus (*E*_c_) values of the sandwich specimens [24].

(1) $$\sigma_{c}=\frac{P_{c}}{A_{c}}$$

(2) $$E_{c}=\frac{m\cdot t}{A_{c}}$$

where *P_c_* is the ultimate load on the compression tests (N); A_c_ is cross sectional area of the sandwich specimens (mm^2^); *m* is the slope of the tangent to the initial straight line portion of the load-deflection curve (N/mm); *t* is the nominal facing thickness (mm).

#### 2.4.2. Three-Point Bending Tests

Three-point bending tests (Figure 3b) were performed according to ASTM C 393 standard [25], with the crosshead speed of 5 mm/min until breaking. For bending tests, 15 specimens were manufactured, 5 for each type of structure core configuration (honeycomb, diamond-celled, corrugated). The radius of punch and supports in the three-point bending was 15 mm, the span length was 110 mm. 

The bending strength (σ_b_) and bending modulus (E_*b*_) values of the sandwich specimens [26] were determined using the following equations:

(3) $$\sigma_{b}=\frac{3\mathrm{PS}}{{2\mathrm{bd}}^{2}}$$

(4) $$E_{b}=\frac{S^{3}m}{{4\mathrm{bd}}^{3}}$$

where P is the force at a given point on the load deflection curve (N); S is the length of support span (mm); b is the sandwich specimen width (mm); d is the sandwich specimen thickness (mm).

The core shear ultimate strength (τ_csu_ (Equation (5))) and facing stress (σ_f_ (Equation (6))) were determined with relations provided by the ASTM C393 standard, specific to three-point bending tested sandwich specimens.

(5) $$\tau_{\mathrm{csu}}=\frac{P}{(d+c)b}$$

(6) $$\sigma_{f}=\frac{\mathrm{PS}}{2t(d+c)b}$$

where c is the core metastructure thickness (mm).

#### 2.4.3. Tensile Tests

**Figure 4:**
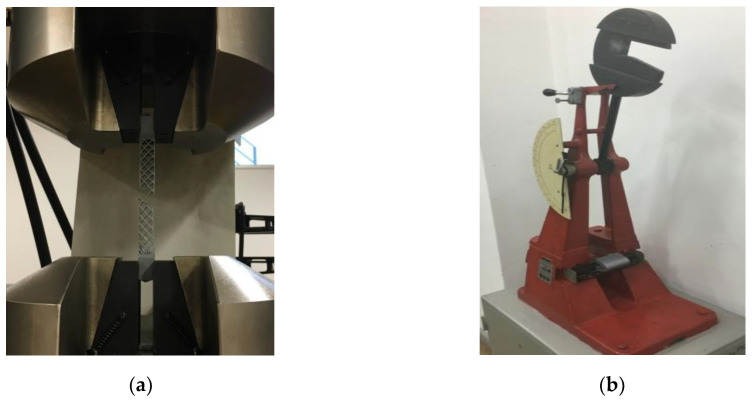
The mechanical tests of the sandwich specimens: (**a**) tensile testing of diamond-celled core sandwich structures; (**b**) charpy impact device.

The method is used to determine the tensile behavior of the sandwich structures and to determine the tensile strength, the tensile modulus and other aspects of the stress–displacement under the defined conditions. The specimens are stressed along its main axis (Figure 4a) at a constant speed of 2 mm/min until breaking according to the conditions of the ASTM D638 standard [27]. For the tensile tests, 15 specimens were manufactured, 5 for each type of core structure configuration (honeycomb, diamond-celled, corrugated).

The tensile strength (*σ*_t_) and tensile modulus (*E*_t_) values of sandwich specimens were calculated using Equation (7) and Equation (8), respectively. Tensile modulus was determined from the slope of stress (*σ*)–strain (*ε*) curves of the sandwich specimens.

(7) $$\sigma_{t}=\frac{P_{t}}{A_{t}}$$

(8) $$E_{t}=\frac{\sigma_{t}}{\varepsilon }$$

*P_t_* is ultimate load (N); A_t_ is the cross sectional area of the sandwich specimen (mm^2^).

#### 2.4.4. Impact Tests

For the three types of core configurations in the structure of the leading edge of the wing impact tests were performed using the Charpy hammer. The initial data were as follows: hammer weight, 6.8 kg; length of pendulum, 380 mm; and the initial potential energy, 49 J. The motivation of these tests comes from the fact that the leading edge of a wing is predisposed to impact with various objects, both in flight and on the ground. Thus, 15 leading edges were tested, 5 for each configuration (honeycomb, diamond-celled, corrugated), having the dimensions of 70 mm length, 45.7 mm width and 50 mm height.

For the specimens with different cores (honeycomb, diamond-celled, corrugated) from the wing leading edges, the Charpy impact strength, *a*_CU_ (kJ/m^2^) was determined by the following relation [28]:

(9) $$a_{\mathrm{CU}}=\frac{E_{c}}{d\cdot b}\cdot 10^{3}$$

where *E_c_* is the energy (J), absorbed by breaking the wing leading edges specimen; d is the thickness (50 mm) of the specimen; and b is the width (45.7 mm) of the specimen.

The device used for testing the impact (Figure 4b) on the sandwich specimens was the Charpy hammer (Web Werkstoffprüfmaschinen, Leipzig, Germany). Double faced adhesive tape was used for fixing the specimens on the Charpy device.

## 3. Results and Discussion

### 3.1. Compressive Performances of Sandwich Specimens

**Figure 5:**
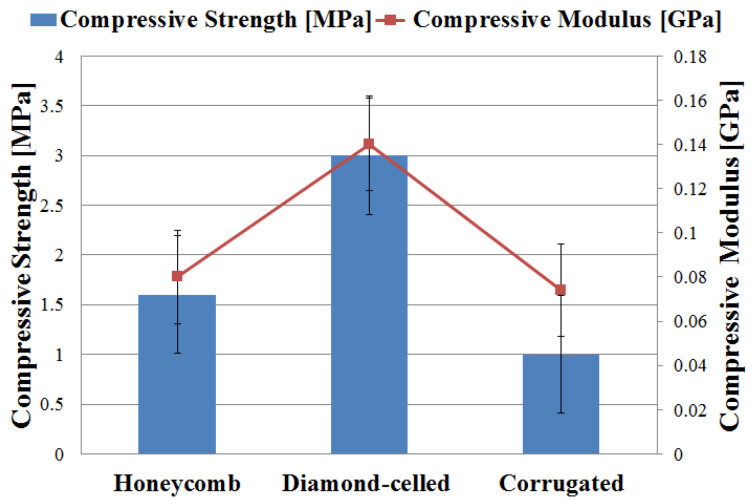
The compressive strength/modulus of the sandwich specimens.

The numerical results shown in Figure 5 represent the average values of the five tests, of compressive stress and compressive modulus. As can be seen from Figure 5, the compressive strength ranges from 1 to 3 MPa, and the compressive modulus falls within the range of 0.074 to 0.14 GPa. 

**Figure 6:**
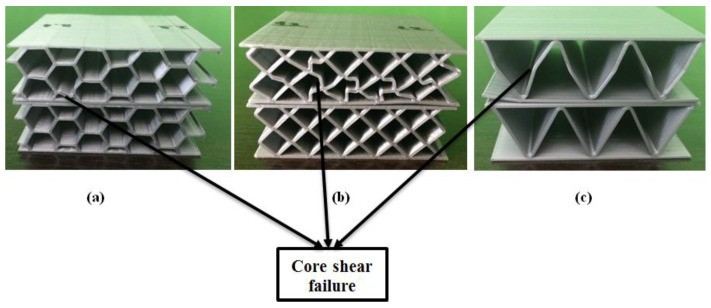
Core shear failures of the sandwich specimens subjected to flatwise compression tests: (**a**) honeycomb core sandwich specimens; (**b**) diamond-celled core sandwich specimens; (**c**) corrugated core sandwich specimens.

The diamond-celled core sandwich structure showed the best performances (compressive strength and compressive modulus) based on experimental tests as compared against the other two types of structures (honeycomb and corrugated), as seen in the Figure 5. The high bending performance of diamond-celled core specimens is due to the dense network of rhombic structures, which also resulted in a higher weight of these specimens. The three types of 3D-printed specimens presented the following mass: 12 g specimens with honeycomb cores; 16 g specimens with diamond-celled cores; 9 g specimens with corrugated cores. There is a very large difference in mass between the specimens, consequently, this variable influenced the entire mechanical analysis. After the compression tests, the upper skin was deformed (Figure 6) on the surface where the punch loaded on the specimen. Deformations appear in the core of the structure which indicates that these sandwich structures will fail locally. The main cause of the structure failure was the shearing of the core sandwich. In the case of sandwich structures subjected to compression tests, the skins were too thick and too strong to be crushed, and therefore a core shear mode will occur.

**Figure 7:**
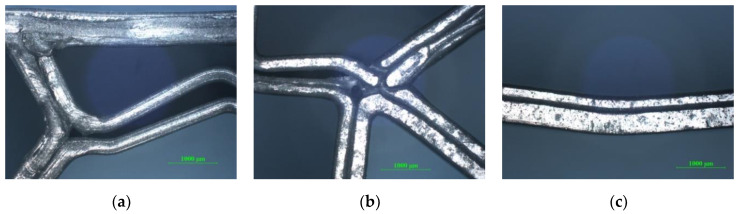
Optical micrographs of failed sandwich specimens subjected to compression tests (magnification 25×): (**a**) honeycomb core sandwich specimens; (**b**) diamond-celled core sandwich specimens; (**c**) corrugated core sandwich specimens.

The optical images of sandwich specimens were taken with a metallographic microscope Nikon Eclipse MA 100 (Nikon Corp., Tokyo, Japan). The specimens shown in Figure 6 were analyzed microscopically. Thus, in Figure 7a,b, a debonding of the extruded filament layers was found, which determined the shearing of the core. In Figure 7c, the shearing of the core did not determine the debonding of the extruded filament layers, because the core has a high flexibility. 

**Figure 8:**
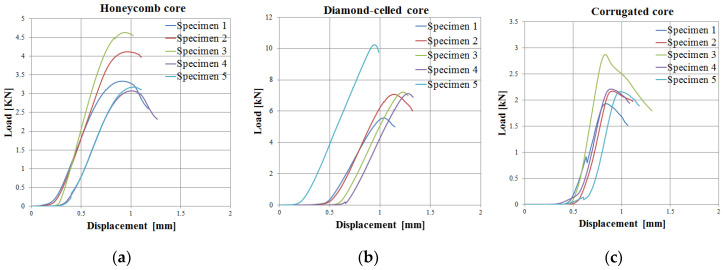
Load–displacement curves obtained from the compressive test of the sandwich specimens: (**a**) honeycomb core; (**b**) diamond-cell core; (**c**) corrugated core.

The graphical representation of the load–displacement is the most used in the experimental study of sandwich structures and obviously describes their mechanical behavior. The load–displacement curves on the compression tests (Figure 8) for each type of cores (honeycomb, diamond-celled, corrugated) show a similar tendency: the load–displacement responses essentially remain linear until the initiation of the core shear, where a sudden drop of load occurs. 

It can be seen that the maximum force, until the irreversible damage in the PLA/PHA material of the sandwich structure had occurred, was approximately 10 kN, in the diamond-cell core structures. Additionally, the displacement at which the irreversible damage occurred in the material of the sandwich structure was 1.3 mm, for the same type of diamond-cell core. The statistical parameters (mean, standard deviation, coefficient of variation) were determined for the sandwich structures with honeycomb, diamond-celled and corrugated core topologies, according to the statistical relationships provided in the ASTM C393 standard, for each data series (compressive strength and compressive modulus). For compressive strength/modulus, the coefficient of variation was determined so as to have a clear image of the homogeneity of the experimental data. The coefficient of variation, as can be seen in Table 5, has values between 5.405% and 10.000% and it can be estimated that the mean is representative for the six sets of experimental data.

### 3.2. Mechanical Characteristics of Sandwich Structures under Three-Point Bending Tests

**Figure 9:**
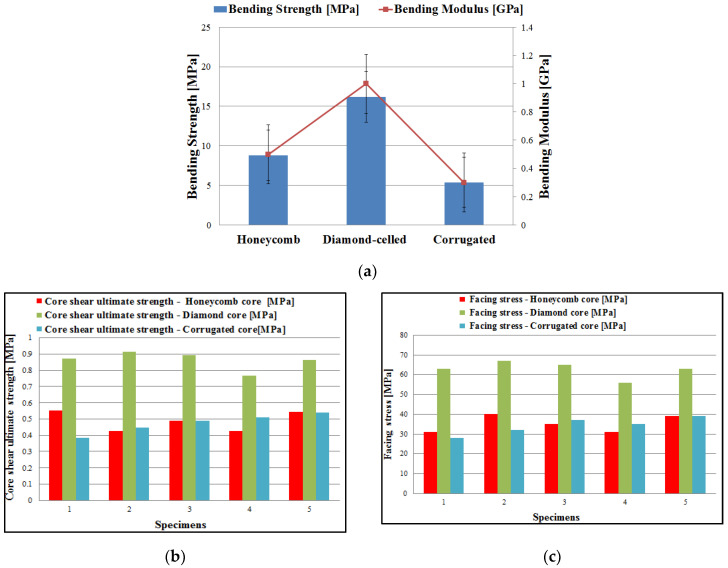
Experimental results: (**a**) the bending strength/modulus of the specimens; (**b**) core shear ultimate strength of the sandwich specimens; (**c**) facing stresses of the sandwich specimens.

This test method is used to investigate the mechanical performance of lightweight sandwich structures (bending strength, bending modulus and stress–displacement aspects). The diamond-celled core sandwich structures showed the best three-point bending results. The corrugated core sandwich specimens had the lowest average bending strength of 5.4 MPa. The diamond-celled core sandwich specimens had an average value of bending strength twice as high as the bending strength of honeycomb core sandwich specimens (Figure 9a). The test machine program automatically calculated the most important three-point bending characteristics (bending strength and the bending modulus), using Equation (3) and Equation (4) and the dimensions of the sandwich specimens. The core shear ultimate strength (τ_csu_) and facing stress (σ_f_) were calculated and plotted in Figure 9b,c, according to ASTM C393 standard. The three types of 3D-printed specimens presented the following mass: 14-g specimens with honeycomb cores; 16-g specimens with diamond-celled cores; 9-g specimens with corrugated cores.

**Figure 10:**
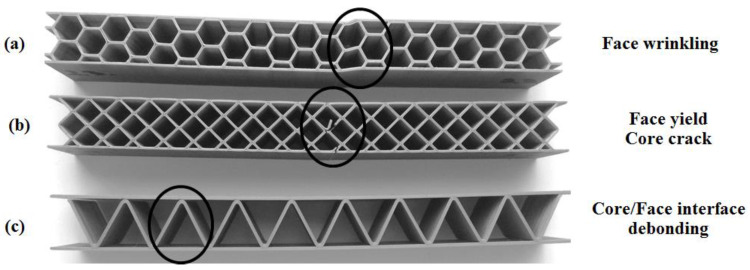
Failure mechanisms of sandwich specimens obtained from the three-point bending test: (**a**) honeycomb core; (**b**) diamond-cell core; (**c**) corrugated core.

Figure 10 describes the main failure modes of sandwich structures subjected to three-point bending tests. The results of the bending tests showed that a failure of tension in the upper skin occurs in this type of sandwich specimen with honeycomb core as compression face wrinkling [29]. 

Face wrinkling failure mode (Figure 10a) is known as local short-wavelength buckling of skins [30]. Because diamond core sandwich skins almost support all the compressive and tensile stresses in bending, sandwich specimens with thin skins may easily fail in a yield mode (Figure 10b). When the core sandwich is thick enough, an indentation mode occurs firstly, but the sandwich structures will finally fail in a face yield failure mode (first phase), of the upper skin, under sufficient impact energy [31]. A second phase consists of the onset of the crack of the diamond core and the subsequent propagation of the crack to the lower skin [32]. The sandwich structures with corrugated core showed a debonding between the skin and the core mainly due to the small contact surface between them (Figure 10c).

**Figure 11:**
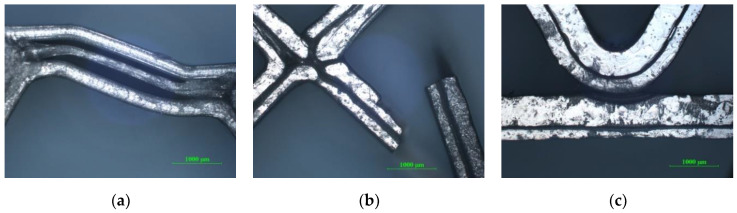
Optical micrographs of failed sandwich specimens subjected to three-point bending tests (magnification 25×): (**a**) Honeycomb core sandwich specimens; (**b**) Diamond-celled core sandwich specimens; (**c**) Corrugated core sandwich specimens.

Figure 11a shows the debonding of the extruded filament layers which determined the failure of the sandwich specimen. For the sandwich specimens with a diamond-celled core (Figure 11b), the complete breakage of the structure appeared during the bending tests. Figure 11.c shows the detachment of the upper skin from the corrugated core of the sandwich structure.

**Figure 12:**
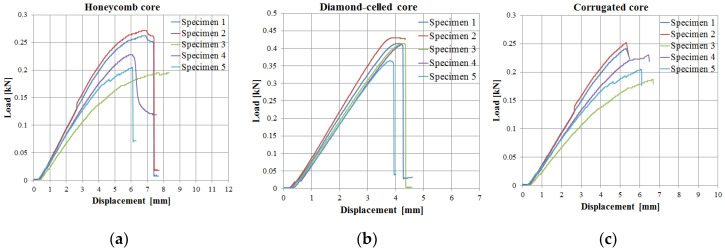
Load–displacement curves obtained from three-point bending tests: (**a**) honeycomb core; (**b**) diamond-cell core; (**c**) corrugated core.

The behavior regarding the load–displacement curves of the 15 specimens tested for three-point bending (Figure 12) presents two main stages: linear increase between the applied load and the displacement with some nonlinear behavior towards the maximum of the curve and then a sudden decrease to maximum load when the specimens broke. Load–displacement curves showed similar shapes for the three types of core studied (honeycomb, diamond-celled, corrugated). After analyzing these curves, it was observed that the maximum force recorded was about 0.428 kN at a displacement of 4.357 mm of diamond-celled core sandwich specimens. 

The high bending performance of diamond-celled core specimens is due to the dense network of rhombic structures, which also resulted in a higher weight of manufactured specimens (12-g specimens with honeycomb cores; 16-g specimens with diamond-celled cores; 9-g specimens with corrugated cores).

The statistical parameters for the bending strengths and bending modulus for the 3D-printed lightweight sandwich with different topologies (honeycomb, diamond-cell and corrugated) are shown in Table 6. The experimental data are homogeneous and the mean is representative because the coefficient of variation of the bending strength/modulus is lower than 30%.

### 3.3. Tensile Behavior of Sandwich Specimens

**Figure 13:**
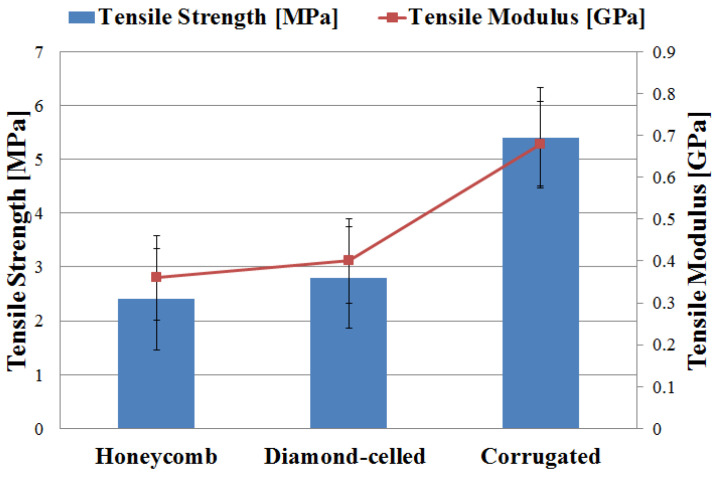
The tensile strength/modulus of the specimens.

Corrugated core sandwich structures showed the best tensile results. The high performances of these types of specimens are due to the corrugated elastic core, which at the tensile stress was almost completely flattened, resulting in higher values of tensile strength and tensile modulus. As it can be seen in Figure 13, the tensile strength ranges between 2.4 and 5.4 MPa, and the tensile modulus falls within the range of 0.36 to 0.68 GPa. The three types of 3D-printed specimens presented the following mass: 18-g specimens with honeycomb cores; 21-g specimens with diamond-celled cores; 17-g specimens with corrugated cores.

**Figure 14:**
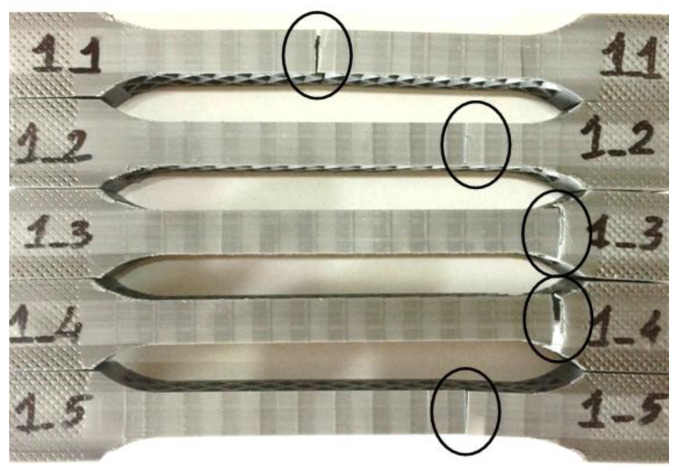
Failure modes of sandwich specimens under tensile load: face sheet yielding and core shear failure.

All the sandwich specimens typically failed through face sheet yielding, followed by core shear failure (Figure 14). The face sheet yielding of the sandwich specimens occurred at the upper face sheet, the crack propagated through the entire core and stopped at the lower level, where it caused a cracking of the lower face sheet.

**Figure 15:**
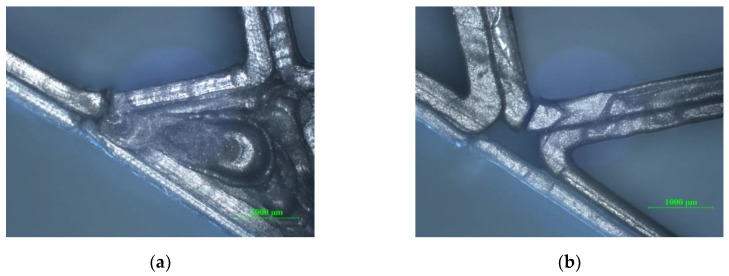
Optical micrographs of failed sandwich specimens subjected to tensile tests (magnification 25×): (**a**) honeycomb core sandwich specimens; (**b**) diamond-celled core sandwich specimens.

As can be seen in Figure 15a,b, the specimens showed a complete breakage of the entire structure starting from the upper skin, the core and finally the lower skin.

**Figure 16:**
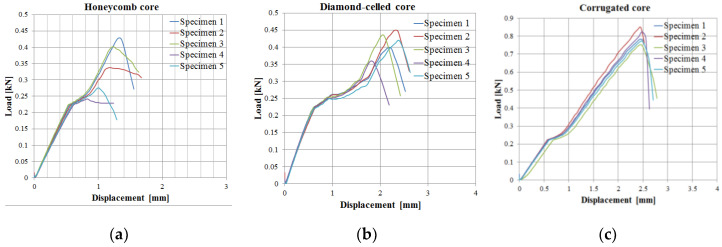
Load–displacement curves obtained from three-point bending tests: (**a**) honeycomb core; (**b**) diamond-cell core; (**c**) corrugated core.

Load–displacement curves, specific to each honeycomb (Figure 16a), diamond-celled (Figure 16b), corrugated cores (Figure 16c), followed the same pattern for all series of sandwich structures. It is well observed that the highest maximum load (0.874 kN) in the load–deflection curves was found for the corrugated core sandwich specimens.

The values of the coefficient of variation (Table 7) of the two sets of experimental data (tensile strength and tensile modulus) is relatively low CV = 10.120% (for tensile strength) and CV = 8.611% (for tensile modulus), so it can be appreciated that the experimental data are homogeneous and the mean is representative.

### 3.4. Strength-to-Mass Ratio Analysis of the Sandwich Specimens

**Figure 17:**
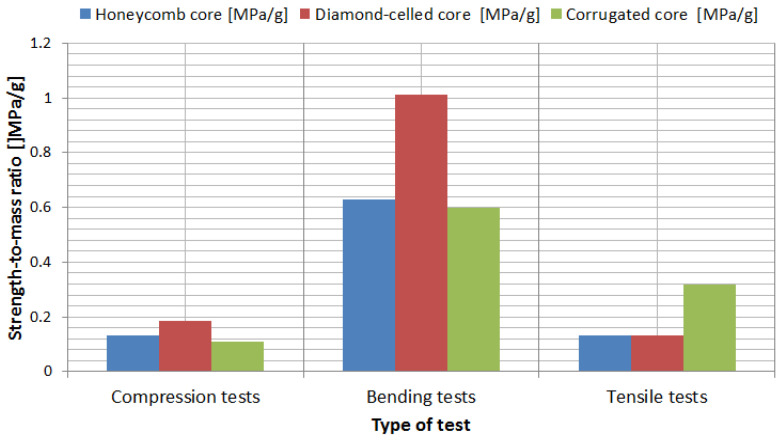
Strength-to-mass ratio analysis of the sandwich specimens.

For a more efficient comparison of the mechanical characteristics of the sandwich structures, a strength-to-mass ratio analysis was used. The strength-to-mass ratio was determined for each type of test (compression, bending and tensile) and also for all three core configurations (honeycomb, diamond-celled and corrugated). Analyzing from the point of view of strength-to-mass ratio (Figure 17), the following conclusions were drawn:

- Based on the compression tests, the sandwich structures with a diamond-celled core presented the best performances;
- Based on the bending tests, the sandwich structures with a diamond-celled core showed the best performances, and the other two core configurations (honeycomb and corrugated) showed similar characteristics;
- Based on the tensile tests, the sandwich structures with a honeycomb core and the sandwich structures with a diamond-cell core showed identical performances, and the sandwich structures with a corrugated core presented the highest performances.

### 3.5. Impact Testing Properties of Wing Leading Edges

**Figure 18:**
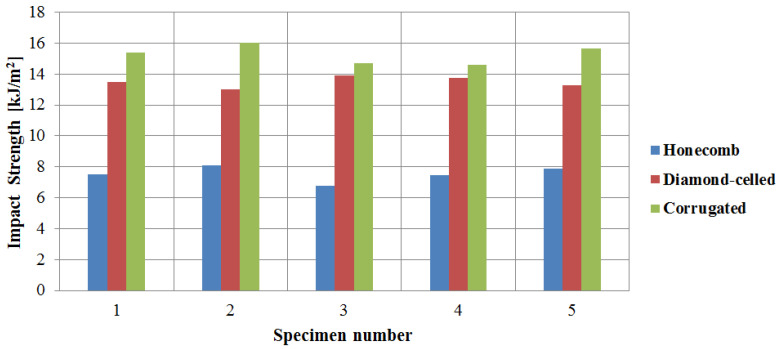
Charpy impact strengths of the wing leading edges specimens.

The Charpy impact strengths for the 15 specimens were determined using Equation (9). Figure 18 describes the calculated Charpy impact strengths, which were between 6.8 kJ/m^2^ (honeycomb core) and 16 kJ/m^2^ (corrugated core). Although honeycomb core specimens and corrugated core specimens had the same weight (32 g), corrugated core specimens exhibit approximately twice the impact resistance, compared to the honeycomb specimens, due to the longitudinal skin-like structures which create increased rigidity. The impact tested specimens showed a complete failure. The breakage was initiated at the level of the specimen skin and propagated throughout the structure.

In the case studies from the engineering field, usually the value of the coefficient of variation has to be between 1% and 30%. Corresponding to the data presented in Table 8, the uncertainty of the set of experimental data (impact strength) is relatively low and shows the values CV = 6.599% and it can be estimated that the mean is representative for the set of experimental data.

### 3.6. Results of Finite Element Analyses

In the simulation model, the dimension of the specimen, the material properties of the specimen, the radius of punch and supports, and the span were set up in agreement with three-point bending tests. In this study, the reaction forces that appear on the sandwich structures using the commercial finite element analysis (FEA) software, ANSYS 19.1 (ANSYS, Inc., Canonsburg, PA, USA) were investigated.

**Figure 19:**
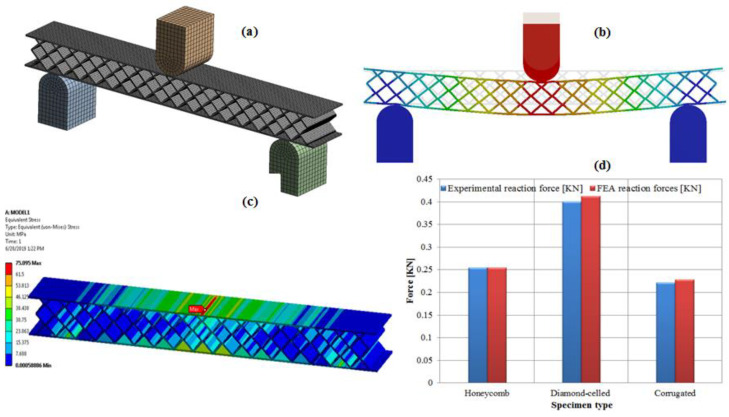
Finite element analysis subject to three-point bending loading: (**a**) finite element analysis (FEA) model—three-point bending; (**b**) deflection of specimen; (**c**) equivalent stress distribution; (**d**) comparative study of reaction forces.

In the finite element analysis, the elastic-plastic model was used for the both components of the sandwich structure (the skin and the honeycomb, diamond-celled, corrugated core). The model was set up and assembled in SolidWorks, and then, was imported in the Ansys 19.1. The sandwich specimen was meshed with fully reduced integration elements (SOLID45 element type), and the mesh size was 0.2 mm (Figure 19a). The punch and supports were meshed with SOLID45 elements as rigid bodies and the mesh size was 0.5 mm. The displacement control method was applied to control the loading step, and the maximum bending deflection of the middle point of specimen was 5 mm (Figure 19b). Friction was considered between the punch, supports and the specimen’s surface and the frictional coefficient was 0.1 [33]. The finite element analysis was carried out following the two aspects: comparative study of the breaking behavior of the three-point bending specimens with that of the finite element analysis of the same specimens; comparative study of the reaction forces that appeared in the breaking of the samples by three-point bending testing and the reaction forces that appeared in the structure of the supports of the FEA model. 

After examining the sandwich specimens with a diamond-cell core subjected to three-point bending tests and those analyzed with finite elements, it can be stated that the breaking occurs, in both cases, at the upper skin of the structure (Figure 19c). The equivalent Von Misses stress presents the maximum value (75.095 MPa) at a middle section on the upper skin of the sandwich structures. Thus, it can be observed that the upper skin of the sandwich specimens, analyzed with FEA, shows the same failure mode as that of the bending tested specimens, namely the face yielding.

The result of the comparative study, between the bending reaction forces resulting from the experimental tests and the reaction forces that appeared in the supports of the FEA model (Figure 19d), presents an adequate validation of the information measured and used when testing the specimens and the FEA model [34], the errors that appear between these results are within a range of 0.4% to 3%.

## 4. Conclusions

Lightweight sandwich structures are extensively used in aviation and automotive industries to reduce the overall weight of the mechanical components. Three different topologies with honeycomb, diamond-celled and corrugated cores were designed and fabricated by fused filament fabrication of PLA/PHA biodegradable material. 

The compressive, three-point bending and tensile properties of sandwich 3D-printed structures with three different core topologies (honeycomb, diamond-celled, corrugated) were investigated. It was found that the diamond-celled core topology presents the highest compression strength and three-point bending strength and the corrugated core exhibited the best tensile strength. The core plays a significant role in the failure of sandwich structures, and after conducting the mechanical tests, the following defects can be distinguished: face yielding, face wrinkling, core shear, and core/skin debonding. 

In order to demonstrate and validate the usefulness of the proposed sandwich structures, the leading edge of the wing segments were developed, with three different topologies (honeycomb, diamond-celled and corrugated cores) using FFF technology. These wing segments were impact tested, and the corrugated core sandwich structure had the highest performance. In addition, the experimental results of the three-point bending tests were validated, making a comparison between the reaction forces that appeared in the experimental tests and the reaction forces of the model analyzed with finite elements, resulting in maximum errors of 3%.

## Figures and Tables

**Table 1:** The proposed lightweight sandwich specimens.

	Compression Test Specimens	Tensile Test Specimens	Three-Point Bending Test Specimens
Honeycomb	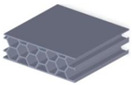	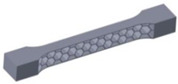	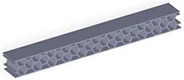
Diamond-celled	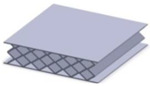	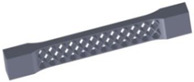	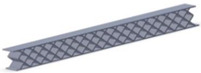
Corrugated	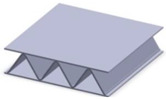	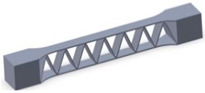	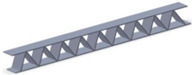

**Table 2:** Wing leading edges specimens.

	Honeycomb Core	Diamond-Celled Core	Corrugated Core
Wing leading edges	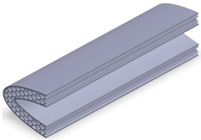	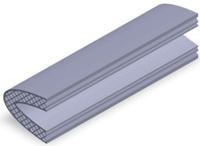	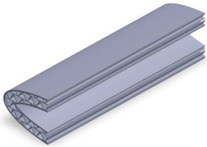

**Table 3:** Mechanical and thermal properties of the material used in 3D printing.

Mechanical and Thermal Properties	PLA/PHA	Standard
Tensile strength [MPa]	61.5	ISO 527-1:2019
Modulus of elasticity [MPa]	2960	ISO 527-1:2019
Flexural stress [MPa]	88.8	ISO 178:2019
Flexural modulus [MPa]	3295	ISO 178:2019
Impact Strength [kJ/m²]	30.8	ISO 179-1:2010
Density [g/cm³]	1.24	ISO 1183:2019
Melting temperature [ºC]	>155	ISO 3146-C:2000

**Table 4:** 3D printing parameters of the specimens.

Parameter	Value
Layer height	0.15 [mm]
Infill	100 [%]
Print speed	50 [mm/s]
Travel speed	200 [mm/s]
Printing temperature	200 [°C]
Building plate temperature	60 [°C]
Hotend	0.4 [mm]

**Table 5:** Statistical parameters determined from the compressive test of sandwich specimens.

Sandwich Specimens	Mean (m)	Standard Deviation (s)	Coefficient of Variation (CV)%
Honeycomb core– Compressive Strength (MPa)	1.600	0.154	9.625
Diamond-celled core– Compressive Strength (MPa)	3.000	0.244	8.133
Corrugated core– Compressive Strength (MPa)	1.000	0.077	7.700
Honeycomb core– Compressive Modulus (GPa)	0.080	0.007	8.750
Diamond-celled core– Compressive Modulus (GPa)	0.140	0.014	10.000
Corrugated core– Compressive Modulus (GPa)	0.074	0.004	5.405

**Table 6:** Statistical parameters determined from the tests at three-point bending of sandwich specimens.

Sandwich Specimens	Mean (m)	Standard Deviation (s)	Coefficient of Variation (CV)%
Honeycomb core– Bending Strength (MPa)	8.800	0.836	9.500
Diamond-celled core– Bending Strength (MPa)	16.200	0.908	5.604
Corrugated core– Bending Strength (MPa)	5.400	0.547	10.129
Honeycomb core– Bending Modulus (GPa)	0.500	0.035	7.000
Diamond-celled core– Bending Modulus (GPa)	1.000	0.079	7.900
Corrugated core– Bending Modulus (GPa)	0.300	0.035	11.666

**Table 7:** Statistical parameters determined from the tensile tests of sandwich specimens.

Sandwich Specimens	Mean (m)	Standard Deviation (s)	Coefficient of Variation (CV)%
Honeycomb core– Tensile Strength (MPa)	2.400	0.200	8.333
Diamond-celled core– Tensile Strength (MPa)	2.800	0.273	9.750
Corrugated core– Tensile Strength (MPa)	5.400	0.547	10.120
Honeycomb core– Tensile Modulus (GPa)	0.360	0.031	8.611
Diamond-celled core– Tensile Modulus (GPa)	0.400	0.028	7.000
Corrugated core– Tensile Modulus (GPa)	0.680	0.024	3.529

**Table 8:** Statistical parameters determined from the impact test of wing leading edges specimen.

Wing Leading Edges Specimens	Mean (m)	Standard Deviation (s)	Coefficient of Variation (CV)%
Honeycomb core– Impact Strength (kJ/m^2^)	7.546	0.498	6.599
Diamond-celled core– Impact Strength (kJ/m^2^)	13.486	0.354	2.624
Corrugated core– Impact Strength (kJ/m^2^)	15.270	0.610	3.994
